# Results of a Randomized, Double-Blinded Trial of Micronutrients and Fish Oil among Patients Diagnosed with Bipolar Disorder

**DOI:** 10.1192/j.eurpsy.2022.1041

**Published:** 2022-09-01

**Authors:** L. Mehl-Madrona

**Affiliations:** Wabanaki Public Health and Wellness, Public Health, Bangor, United States of America

**Keywords:** bipolar disorder, Micronutrients, Fish Oil, Randomized, controlled trial

## Abstract

**Introduction:**

In a previous open-label study, we found that patients with bipolar disorder improved in symptom level when taking micronutrients and fish oil. We planned a randomized, double-blind, controlled trial to explore the feasibility and parameters needed for a larger clinical trial.

**Objectives:**

We aimed to determine the parameters necessary to conduct a large-scale clinical trial through completing a feasibility study.

**Methods:**

Patients were screened for having the diagnosis of bipolar disorder and being willing to take up to 16 micronutrient capsules and 3 fish oil capsules per day. Patients were randomized in a 3:2 ratio to micronutrients or placebo. Patients were seen monthly with assessment of the Clinical Global Impression Scale, the UKU Side Effects Scale, and a review of their medication doses. On a quarterly basis, patients completed the BASIS-24, the MYMOP-2, the Young Mania Scale, and the MADRS questionnaire

**Results:**

The setting was a primary care clinic in Maine in the United States. The patient population was low-income and primarily rural. Disease severity was mild to moderate as only 2 patients were hospitalized during the study. All were symptomatic. One hundred twenty-five patients were screened and accepted randomization. The attrition rate was high and only 52 subjects completed 6 months of treatment. No differences were found between the two groups. We calculated that a minimum of 250 subjects would be needed to have 80% power to detect a difference. All patients improved dramatically in all measures.

**Conclusions:**

Bipolar patients in primary care remain moderately symptomatic and will improve dramatically with monthly visits.

**Disclosure:**

No significant relationships.

